# Estimating the benefit of esketamine nasal spray versus real-world treatment on patient-reported functional remission: results from the ICEBERG study

**DOI:** 10.3389/fpsyt.2024.1459633

**Published:** 2024-10-07

**Authors:** Albino J. Oliveira-Maia, Benoît Rive, Yordan Godinov, Siobhán Mulhern-Haughey

**Affiliations:** ^1^ Champalimaud Research and Clinical Centre, Champalimaud Foundation, Lisbon, Portugal; ^2^ NOVA Medical School, Faculdade de Ciências Médicas, NMS, FCM, Universidade NOVA de Lisboa, Lisbon, Portugal; ^3^ Janssen EMEA, Paris, France; ^4^ Janssen EMEA, Sofia, Bulgaria; ^5^ Janssen EMEA, Dublin, Ireland

**Keywords:** treatment resistant depression, patient-reported outcome, indirect treatment comparison, functional remission, esketamine nasal spray, functioning, Sheehan Disability Scale

## Abstract

**Introduction:**

Treatment resistant depression (TRD) affects approximately 10–30% of patients with major depressive disorder, and most patients with TRD do not respond to real-world treatments (RWT). Treatment with esketamine nasal spray (NS) plus a selective serotonin or serotonin norepinephrine reuptake inhibitor (SSRI/SNRI) has significant long-term clinical benefit over RWT in patients with TRD. However, the impact on patient-reported function remains to be determined.

**Methods:**

The ICEBERG analysis was an indirect treatment comparison performed using data from two studies of patients with TRD: SUSTAIN-2 (esketamine NS; NCT02497287) and the European Observational TRD Cohort (EOTC; RWT; NCT03373253; clinicaltrials.gov). Here, patient−reported functional remission, assessed using the Sheehan Disability Scale (SDS), was defined as SDS ≤6 at Month 6. Analyses were conducted using propensity score re−weighting and multivariable models based on 18 covariates.

**Results:**

At Month 6, the probability of functional remission in esketamine NS−treated patients from SUSTAIN-2 (n=512) was 25.6% (95% confidence interval [CI] 21.8–29.4), while the adjusted probability for RWT patients from the EOTC (n=184) was 11.5% (95% CI 6.9–16.1; relative risk: 2.226 [95% CI 1.451–3.416]; p=0.0003). In the total combined population (N=696), patients who did not achieve clinical response or remission had a low probability of achieving functional remission (5.84% and 8.76%, respectively). However, for patients who did achieve clinical response or remission, the probability of achieving functional remission was greater (43.38% and 54.15%, respectively), although many still did not achieve this status.

**Conclusions:**

For patients with TRD, esketamine NS had a significant functional benefit versus RWT after 6 months of treatment. Irrespective of treatment, achievement of clinical response or remission was insufficient to attain functional remission. Nevertheless, clinical remission increased the likelihood of achieving functional remission, further supporting an important role for clinical remission in for the path towards functional recovery.

## 1 Introduction

Treatment resistant depression (TRD), commonly defined as a major depressive episode that fails to respond to two or more antidepressants, affects approximately 10–30% of patients with major depressive disorder (MDD) (1–6). Patients with TRD face high rates of functional impairment and reduced health−related quality of life in association with symptoms of depression (3, 7). Even patients who achieve clinical response to treatment do not experience the same level of functional improvements as those who achieve clinical remission, and may continue to have functional impairments such as difficulty performing self-care or completing housework (1, 8). Patients with TRD also experience higher economic costs versus patients who are treatment responsive, due to productivity loss and workplace impairment (9). Patient−reported functional remission is thus an important treatment goal that should be assessed in addition to clinical response or remission, as it is unclear whether antidepressant treatment significantly improves measures of workplace functioning (10). One method to determine functional impairment is the Sheehan Disability Scale (SDS), a patient−reported outcome (PRO) of functional disability, assessing disruption of several aspects of daily life, including impacts on work and/or school work, social life and leisure activities, and family life and home responsibilities (11, 12). Decreases in SDS scores indicate an improvement of daily functioning, and can be used to determine rates of functional remission.

Real−world treatment (RWT) for TRD may include any treatment or combination of treatments approved for use in MDD (1, 8). Indeed, there is no consensus on the standard of care for TRD, with typical treatments ranging across a large spectrum of options. Treatment choice is influenced by many aspects, including treatment pathway stage (acute, continuation or maintenance) and the severity of the patient’s depressive symptoms (1, 13). Treatment options often include monotherapy with an antidepressant (e.g. selective serotonin reuptake inhibitors [SSRI] or serotonin norepinephrine reuptake inhibitors [SNRI]), a combination of antidepressants, or augmentation with an antipsychotic or mood stabiliser (8). Augmentation of an SSRI/SNRI with quetiapine extended release is one of many available options to treat TRD in the real world (14). A more recent option for TRD is esketamine, an N−methyl−D−aspartate (NMDA) receptor antagonist, which is the only treatment specifically approved for TRD in Europe (15). Esketamine nasal spray (NS), in combination with a SSRI or SNRI, has been shown to be superior to placebo plus SSRI/SNRI over a 4-week period, and superior to quetiapine extended release plus SSRI/SNRI over an 8-week acute period and a 24-week maintenance period (15–20). Thus, there is a need to compare esketamine NS with RWT both for short− and long−term treatment periods.

The Indirect adjusted Comparison Estimating the long−term Benefit of Esketamine NS when compared with Routine treatment of TRD in General psychiatry (ICEBERG) was the first comparison of long−term esketamine NS with RWT. In ICEBERG, analyses of data from two previous studies of TRD treatment were performed. Since these studies were conducted in similar circumstances, this allowed for the indirect comparison. Previous ICEBERG analyses demonstrated that patients with TRD receiving esketamine NS were almost twice as likely to achieve clinical response or remission when compared with patients under RWT at 6 months (21, 22). However, these previous analyses did not assess functional remission in patients with TRD. Here, our objective was to leverage ICEBERG to compare functional remission rates between patients receiving esketamine NS and those receiving RWT at 6 months.

## 2 Methods

The goal of the ICEBERG analyses was to mimic a hypothetical randomised trial comparing the treatment effect of esketamine NS (from SUSTAIN-2) and RWT (from the European Observational TRD Cohort [EOTC]). The potential range of treatments in the EOTC may have introduced complexities in estimating treatment effects, which this methodology aims to address.

### 2.1 Study designs

ICEBERG was performed using individual patient data from the first 6 months of two studies of patients with TRD. SUSTAIN−2 (NCT02497287) was an open−label, long−term global study that evaluated the safety and efficacy of esketamine NS plus a newly initiated SSRI/SNRI in patients with TRD (23). The EOTC (NCT03373253) was a prospective, non−interventional, multi−centre study of patients initiating a new, routine treatment for TRD in real−world clinical practice, prior to approval of esketamine NS (1). All patients in the EOTC were receiving medication and/or other treatments according to usual care in their treatment setting (1, 24).

In both the EOTC and SUSTAIN-2 studies, patients were allowed full flexibility regarding psychotherapy and could continue, initiate and/or stop psychotherapy at any point, as deemed necessary by the study physician. However, data related to psychotherapy were not able to be included in this analysis due to a lack of structured data collection in SUSTAIN-2. Some neuromodulatory treatments were allowed in the EOTC only, but were used only in a minority of patients (6.6%), and were thus also not included in analyses (1).

These studies were selected for comparison due to similarities in inclusion and exclusion criteria, definition of TRD and long−term follow up of patients. More detail regarding the individual studies can be found in previous ICEBERG publications (21, 22), and the study−specific publications for SUSTAIN−2 and the EOTC (1, 23). All participants in SUSTAIN-2 and the EOTC provided written informed consent.

### 2.2 Indirect treatment comparison

This indirect treatment comparison (ITC) included data from patients starting esketamine NS in addition to an SSRI/SNRI from SUSTAIN−2 and from patients starting a treatment involving at least one oral antidepressant medication from the EOTC.

All direct−entry patients recruited for SUSTAIN-2 were considered for ICEBERG while patients that entered SUSTAIN−2 from the TRANSFORM−3 phase 3 trial were excluded from analysis. Patients who did not reach a treatment response at Week 4 of SUSTAIN-2 did not proceed to the study’s maintenance phase but were nevertheless included in the ICEBERG analysis. These patients were assumed not to reach functional remission and were included through non−responder imputation (NRI) for analyses of clinical response, clinical remission and functional remission. An equivalent approach was used for patients who dropped out from SUSTAIN-2 before Month 6. However, the SUSTAIN−2 study design included a provision of study termination after reaching a predetermined recruitment target, which resulted in less than six months of study enrolment for some patients. For these patients, 6-month data could not be collected and, since non-responder imputation was not considered appropriate, they were excluded from ICEBERG analyses.

For the EOTC, patients who did not receive antidepressant medication as part of their first treatment within the study (i.e., patients treated only with neuromodulation, psychotherapy or with an antipsychotic or mood stabiliser as monotherapy) were excluded from the analysis. Since esketamine NS was not available for prescription during the EOTC, no patient in the EOTC received this medication. As the EOTC was terminated when the last patient reached the 6−month follow up visit, exclusion due to study termination was not necessary. Patients who switched or added treatments during the 6−month period were included in the ICEBERG analysis, given that this reflects RWT, as is the objective of the study. However, drop−outs from the EOTC were considered non−informative and were excluded from the ICEBERG analysis, given that a change in treatment site is not infrequent in RWT and does not imply lack of response or remission.

Patient−reported functioning was assessed using the SDS, which measures the impact of disability or illness on a patient’s daily life in three domains: work and/or school work, social life and leisure activities, and family life and home responsibilities (11, 12). Each domain is scored on a 0–10 scale, with the total SDS score being the sum of the work/school score, the social life/leisure score and the family life/home responsibilities score. The maximum total score is 30, with higher scores representing a greater impact of illness on patients’ daily functioning. Functional remission was defined as a total SDS score of ≤6 at Month 6 (25), both for patients receiving esketamine NS plus SSRI/SNRI and for patients receiving RWT. Non-working patients were excluded from the analysis as the work item score of the SDS could not be assessed; no imputation for non-working patients was performed. These treatment groups were also pooled, for comparisons between functional remission and clinical remission (total MADRS score ≤10) as well as clinical response (≥50% improvement in total MADRS score compared to baseline). Clinical remission and clinical response data have been reported in previous ICEBERG publications (21, 22).

### 2.3 Statistical analysis

ICEBERG was not a randomised comparison, but rather an ITC, which required strategies for adjustment of analyses (21, 22). To account for potential bias and the effect of observed confounders, a propensity score (PS)-based inverse probability weighting (IPW) was applied based on 18 covariates. Covariates included patient characteristics at baseline including demographics and clinical scores (**Supplementary Table 1**). Treatment differences were estimated using a rescaled average treatment effect among treated (ATT) IPW method (26), where patients in the EOTC were reweighted according to the PS distribution from SUSTAIN-2 (as if patients had been randomised between the two arms). Weights were rescaled to correspond to the original number of patients (by re−weighting observations in the EOTC with SUSTAIN−2 as the reference). To avoid artificial inflation in sample size, patients in SUSTAIN-2 all had a weight of one. The re−weighted EOTC data acted as a synthetic control arm for SUSTAIN−2.

Outputs were probabilities of achieving functional remission for each treatment, and the efficacy measures of odds ratios (OR), relative risk (RR) and risk differences (RD), along with their respective 95% confidence intervals (CI). The number needed to treat (NNT) is also reported and is derived from the RD. All outputs were estimated using weighted logistic regression. The ability of reweighting to reduce potential imbalances between studies was assessed by comparing the weighted distribution of PS of the reweighted populations and the standardised mean difference (SMD) of each covariate between the two studies before and after reweighting. SMD values between –0.2 and +0.2 indicate that differences would not be clinically detectable (27).

When statistically significant differences between the two studies were observed, threshold analyses were carried out. Simulations were performed in which the functional remission rate in the esketamine NS arm (SUSTAIN-2) was progressively decreased, while keeping the functional remission rate in the RWT arm (EOTC) unaltered. At each iterative rate decrease, the main analysis was replicated to check if statistical significance was maintained. This was performed separately for each efficacy indicator (OR, RR and RD). Differences between observed and simulated results were computed to understand how much lower functional remission rates in the esketamine NS arm could have been while still showing statistically significant superiority versus RWT. Results from these threshold analyses were further illustrated by examining to what extent conclusions from the main analyses would be preserved in the presence of a hypothetical unobserved confounder that would be unbalanced between treatment arms and have an impact on main outcomes.

### 2.4 Multivariable analysis

A multivariable logistic regression model, including the same 18 covariates and using pooled individual patient data from both studies, was also used to compare esketamine NS and RWT. This allowed estimation of the adjusted OR to quantify the relative treatment effect and accounted for imbalances between cohorts. Variables were included in the analysis sequentially by rank (**Supplementary Table 1**).

## 3 Results

### 3.1 Patient disposition and baseline characteristics

Study flow diagrams for patients included in ICEBERG can be found in **Supplementary Figure 1**. Before reweighting, baseline characteristics were similar across studies (**Table 1**). For esketamine NS−treated patients (n=512), mean (standard deviation [SD]) age was 48.8 years (12.4), SDS total score was 22.5 (5.0), MADRS total score was 31.3 (5.0), number of episodes was 4.1 (3.4), duration of current episode was 132.6 weeks (223.8) and number of treatment failures in the current MDE was 2.6 (1.0). For patients receiving RWT (n=184), mean (SD) age was 50.2 years (9.9), SDS total score was 21.9 (5.5), MADRS total score was 32.0 (5.9), number of episodes was 3.8 (3.6), duration of current episode was 131.9 weeks (180.1) and number of treatment failures in the current MDE was 2.6 (0.9). While there was some overlap in PS before reweighting, there were differences between studies. After reweighting, the PS distributions were similar (**Supplementary Figure 2**) and SMDs were generally reduced (**Table 1**, **Supplementary Table 2**). Of the SMDs for each of the 18 covariates, 14 fell between +0.2 and –0.2 after reweighting, versus 9/18 before reweighting.

**Table 1:** Baseline demographics and patient characteristics.

Mean (SD)	Esketamine NS^a^ (n=512)	RWT (n=184)
Age, years	48.8 (12.4)	50.2 (9.9)
Age at diagnosis, years	34.3 (13.0)	37.5 (13.0)
Time since diagnosis, years	14.5 (11.2)	12.7 (11.0)
Number of episodes	4.1 (3.4)^b^	3.8 (3.6)^c^
Duration of current episode, weeks	132.6 (223.8)	131.9 (180.1)
Average treatment duration, weeks	43.3 (69.8)	52.5 (74.5)
Number of treatment failures in the current MDE	2.6 (1.0)	2.6 (0.9)
SDS total score	22.5 (5.0)	21.9 (5.5)
MADRS total score	31.3 (5.0)	32.0 (5.9)
CGI−S score	4.9 (0.7)	4.8 (0.8)
EuroQoL VAS score	44.3 (19.8)	40.7 (18.5)^c^

^a^Esketamine NS was taken in addition to an SSRI/SNRI. ^b^Data missing for one patient. ^c^Data missing for two patients. CGI−S, Clinical Global Impressions−Severity; EuroQoL, European Quality of Life; MADRS, Montgomery−Åsberg Depression Rating Scale; MDE, major depressive episode; NS, nasal spray; RWT, real−world treatment; SD, standard deviation; SDS, Sheehan Disability Scale; SMD, standardised mean difference; SNRI, serotonin−norepinephrine reuptake inhibitor; SSRI, selective serotonin reuptake inhibitor; VAS, visual analogue scale.

### 3.2 Predicted probability of functional remission

**Figure 1:**
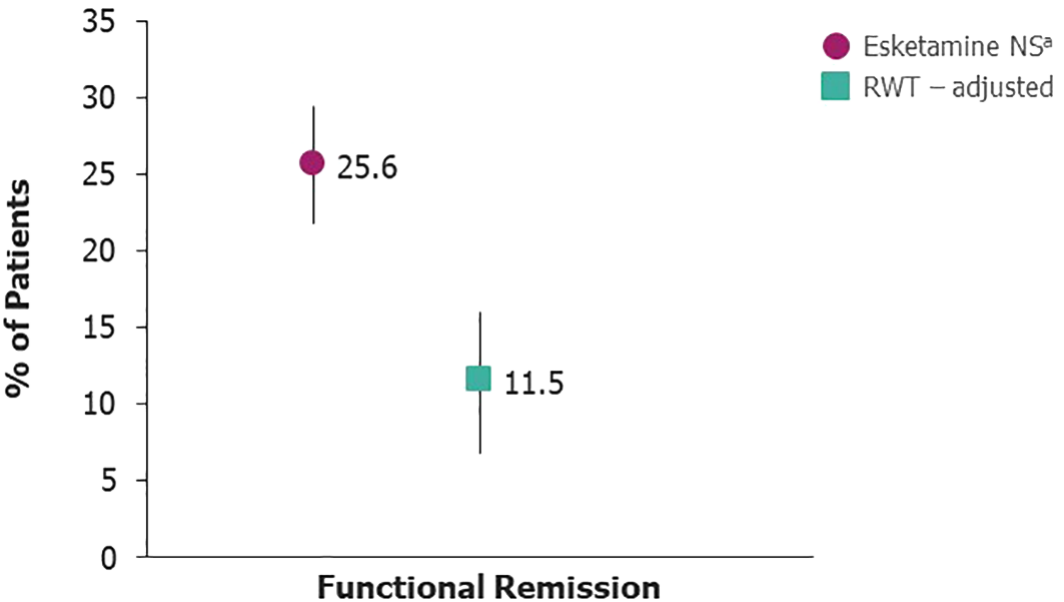
Probability of functional remission. ^a^Esketamine NS taken in addition to an SSRI/SNRI. NS, nasal spray; RWT, real−world treatment; SNRI, serotonin−norepinephrine reuptake inhibitor; SSRI, selective serotonin reuptake inhibitor.

At Month 6, the probability of functional remission was 25.6% (95% CI 21.8–29.4) for esketamine NS−treated patients (**Figure 1**). The adjusted probability for patients receiving RWT was 11.5% (95% CI 6.9–16.1). The unadjusted probability was 12.5% (95% CI 7.7–17.3).

When comparing esketamine NS with RWT, the OR for functional remission at Month 6 was 2.648 (95% CI 1.613–4.346; p=0.0001), the RR was 2.226 (95% CI 1.451–3.416; p=0.0003) and the RD was 0.141 (95% CI 0.081–0.201; p<0.0001). The NNT was 8 (95% CI 5–13) (**Table 2**).

**Table 2:** Chance of achieving functional remission at Month 6.

Esketamine NS^a^ vs RWT	Result (95% CI)	p value
OR	2.648 (1.613–4.346)	0.0001
RR	2.226 (1.451–3.416)	0.0003
RD	0.141 (0.081–0.201)	<0.0001
NNT	8 (5–13)	N/A

^a^Esketamine NS was taken in addition to an SSRI/SNRI. RWT data were adjusted using the ATT covariate adjustment method. OR>1, RR>1 and RD>0 all indicate that esketamine NS is superior to the comparator treatment. ATT, rescaled average treatment effect among treated; CI, confidence interval; N/A, not applicable; NNT, number needed to treat; NS, nasal spray; OR, odds ratio; RD, risk difference; RR, relative risk; RWT, real−world treatment; SNRI, serotonin−norepinephrine reuptake inhibitor; SSRI, selective serotonin reuptake inhibitor.

### 3.3 Multivariable analysis of functional remission

**Figure 2:**
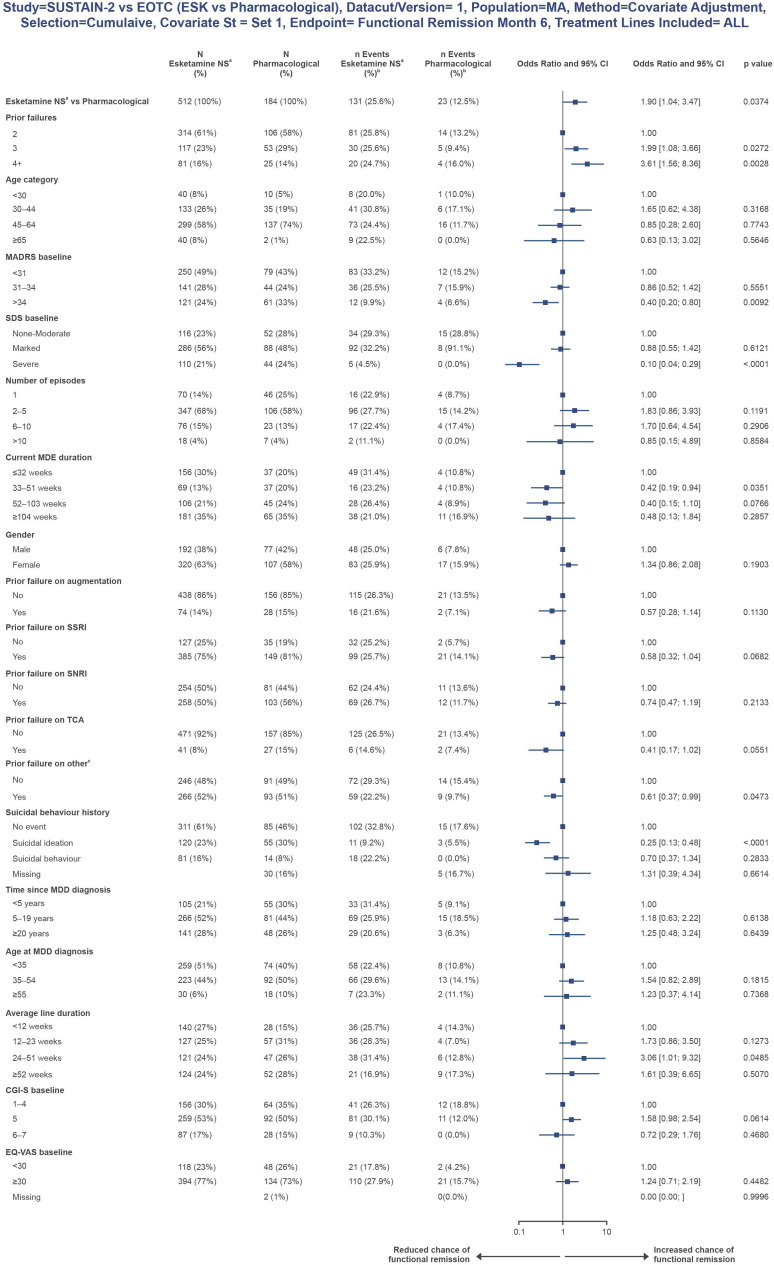
Multivariable logistic regression plot for 6-month functional remission. Naïve 6-month functional remission plot. RWT excludes esketamine NS. ^a^Given in combination with an SSRI or SNRI; ^b^This percentage was computed as number of events/N; ^c^Prior failure on ‘other’ included trazodone, nefazodone, vilazodone, bupropion, mirtazapine, mianserin, opipramol, agomelatine, tianeptine, reboxetine and vortioxetine. CGI-S, Clinical Global Impression-Severity; CI, confidence interval; EQ-VAS, EuroQol Visual Analogue Scale; MADRS, Montgomery-Åsberg Depression Rating Scale; MDD, major depressive disorder; MDE, major depressive episode; NS, nasal spray; OR, odds ratio; RWT, real-world treatment; SNRI, serotonin-norepinephrine reuptake inhibitor; SSRI, selective serotonin reuptake inhibitor; TCA, tricyclic antidepressant.

Covariates were included in the analysis sequentially by rank, and all were included. After adjustment of all 18 baseline covariates, the OR for 6-month functional remission favoured esketamine NS over RWT (OR: 1.90 [95% CI 1.04–3.47]; p=0.0374; **Figure 2**).

### 3.4 Threshold analyses

Threshold analyses showed that a 7.6–8.4% reduction in the functional remission rate could occur in patients receiving esketamine NS before loss of significance in comparison with RWT (loss of significance was p≥0.05; **Table 3**), depending on the efficacy measure.

**Table 3:** Threshold analysis based on OR, RR and RD for chance of 6−month functional remission.

	Probability in esketamine NS arm, % (95% CI)		
Efficacy measure	Observed	Lowest significant simulated result^a^	Difference,^b^ %
OR	25.6 (21.8–29.4)	17.8 (14.5–21.1)	7.8
RR	25.6 (21.8–29.4)	18.0 (14.6–21.3)	7.6
RD	25.6 (21.8–29.4)	17.2 (13.9–20.5)	8.4

^a^Pre−determined significance value was p<0.05. ^b^Maximum difference in functional remission before loss of significance in outcomes. CI, confidence interval; OR, odds ratio; RD, risk difference; RR, relative risk.

### 3.5 Chance of achieving functional remission based on clinical outcome

Of the total study population (patients pooled from both SUSTAIN-2 and the EOTC; N=696), patients who did achieve clinical remission had a 54.15% chance of achieving functional remission, and patients who achieved clinical response had a 43.38% chance of achieving functional remission (**Table 4**). Patients who did not achieve clinical remission had an 8.76% chance of functional remission. Patients who did not achieve clinical response had a 5.84% chance of functional remission (**Table 4**).

**Table 4:** Chance of achieving functional remission at Month 6 based on clinical outcome for the total population (N=696).

	Functional remission achieved	No functional remission
No clinical remission	8.76% (n=43)	91.24% (n=448)
Clinical remission achieved	54.15% (n=111)	45.85% (n=94)
No clinical response	5.84% (n=23)	94.16% (n=371)
Clinical response achieved	43.38% (n=131)	56.62% (n=171)

## 4 Discussion

Previous analyses from ICEBERG have demonstrated that patients with TRD receiving esketamine NS were almost twice as likely to achieve clinical response or remission when compared with RWT over 6 months (21, 22). The ITC analysis presented here further suggests esketamine NS has a significant functional benefit at Month 6 compared with RWT for patients with TRD. The probability of achieving functional remission for patients who received esketamine NS was significantly higher than the estimated probability for patients who received RWT. All efficacy measures (OR, RR, RD) indicated the benefit of esketamine NS over RWT was statistically significant. The NNT was in also in favour of treatment with esketamine NS.

Functional remission was considered the point where depressive symptoms no longer have a substantial detrimental impact on a patient’s daily functioning, as per an SDS score of ≤6. The mean baseline SDS scores for esketamine NS-treated patients and patients treated with RWT were 22.5 and 21.9, respectively. These values, scored from 0 to 30, indicate the marked functional impairment experienced by patients and the importance of considering functional remission as a treatment goal. Indeed, for the overall population included in this analysis (n=696), functional remission is more difficult to achieve than clinical response or remission, with fewer patients achieving functional remission (n=154, 22.1%) versus clinical remission (n=205, 29.5%) and clinical response (n=302, 43.4%; **Table 4**) (21). Furthermore, the chance of achieving functional remission was higher in patients who achieved clinical remission or clinical response compared with those who did not. As patients that do not achieve clinical response or clinical remission have low chances of achieving functional remission, these analyses demonstrate a notion of the increasing difficulty of achieving these endpoints at an individual patient level. It is worth noting that this analysis could not be evaluated by treatment arm due to factors that happen after baseline and the pseudo-randomisation. These results from the overall population suggest that clinical response and clinical remission are necessary first steps towards achieving functional remission. Indeed, our findings support that clinical remission should be the goal of treatment, as it provides patients with the best chance of improvements in day−to−day functioning.

The ITC methods used for these analyses are widely accepted and are used when a direct comparison is lacking (28–33). PS re−weightings were used to rule out potential bias between the two populations. Indeed, after reweighting, a greater proportion of SMDs were between –0.2 and +0.2 indicating that these differences would not be clinically detectable (**Supplementary Table 2**) (27). Results from adjusted versus unadjusted comparisons were largely similar (for example, the adjusted probability of achieving functional remission for patients receiving RWT was 11.5%, while the unadjusted probability was 12.5%), suggesting any differences in patient characteristics between study populations had no major impact on the findings. Following adjustment for the 18 covariates, consistent results demonstrate the robustness of the comparison; as covariates were progressively introduced, the treatment effect was relatively stable as demonstrated by overlapping confidence intervals and significant p values at each step.

The measure of functional remission used in these analyses was derived from the SDS (11, 12), which includes questions on work and/or school, social life and family life. Individuals who were not able to work could not answer the work section of the SDS, which led to the exclusion of patients who were not working from this analysis. Exclusion of these highly impaired patients is a limitation of this analysis, since it may have resulted in an overestimation of functional remission. However, since missing SDS scores were more prominent in the RWT population, it is unlikely that comparative analyses of likelihood of functional remission versus RWT were overestimated. Exclusion of these patients was also the reason why the total number of patients eligible for inclusion in this analysis was lower than the previous ICEBERG analyses (21, 22). For the previous analyses on clinical remission and response, there were 559 patients on esketamine NS and 307 on RWT (21, 22). For this functional remission analysis, there were 512 and 184, respectively.

In addition to the exclusion of non-working patients, patients with a psychotic disorder, MDD with psychotic features and/or bipolar disorder, patients with a history of substance abuse/misuse and patients with recent suicidal ideation with some intent to act were excluded from SUSTAIN-2 and the EOTC. This limits the generalisability of these results in these populations, however, studies on esketamine NS treatment in these subpopulations were consistent with results presented here (34, 35).

A final limitation of this study was that strategies for re−weighting and adjustment could only be performed for observed patient characteristics, and it is therefore still possible that unobserved characteristics may have been confounders. Exploratory threshold analyses were carried out to assess the possible impact of these potential confounders. Depending on the efficacy measure, if 7.6%–8.4% fewer esketamine NS patients achieved functional remission, esketamine NS would still maintain statistical significance over RWT. An alternative way to interpret the threshold analyses is to determine the impact of a potential (unobserved) confounder. If a potential unobserved confounder existed that was 25% more prevalent in SUSTAIN−2 and this confounder increased the chance of functional remission by 30%, it would increase the functional remission rate for patients in SUSTAIN-2 by 7.5% (25%*30%=7.5%). This would still fall within the threshold of statistical significance, as this value is less than 7.6% (the lower boundary of the threshold analysis). It would be unlikely for such a cofounder to exist. Further detail on the methodology, and discussion on the strengths and limitations of the ITC can be found in previous ICEBERG publications (21, 22).

Other randomised and non−randomised studies support the superiority of esketamine NS in reaching treatment goals that are important to patients. Previous ICEBERG analyses comparing esketamine NS to the total population and to patients treated with polypharmacy strategies (combination and augmentation therapies) supported the superiority of esketamine NS for clinical remission and response (21, 22). Additionally, primary results from ESCAPE−TRD (NCT04338321), a randomised controlled trial comparing esketamine NS with quetiapine extended release, show that a significantly greater proportion of esketamine NS−treated patients achieve remission at Week 8, and remission at Week 8 with no relapse up to Week 32, when compared with those treated with quetiapine extended release (20).

This ICEBERG analysis is the first to report a comparison of long-term results of treatment with esketamine NS relative to a heterogenous mix of RWT in patients with TRD for achievement of functional remission. Additional analyses supported that clinical response and clinical remission are the first steps towards achievement of functional remission. Over a 6-month period, treatment with esketamine NS resulted in a higher proportion of patients achieving clinical response and clinical remission, but also a greater probability of patient−reported functional remission, when compared with other RWT. The robustness of the ITC is supported by PS re-weighting, adjusted analyses and threshold analyses. Our findings support that esketamine NS is a more effective alternative compared to the current standard of care to help patients with TRD achieve meaningful, functional remission.

## Data Availability

The datasets presented in this article are not readily available because Janssen EMEA’s Data Sharing Policy does not include non-interventional studies, of which this analysis is included. However, if you are interested in the SUSTAIN-2 data, requests for access to these study data can be submitted through the Yale Open Data Access (YODA) project site at http://yoda.yale.edu. Requests to access the datasets should be directed to brive@its.jnj.com.
