# Supplementary material for: Estimating the benefit of esketamine nasal spray versus real-world treatment on patient-reported functional remission: results from the ICEBERG study
Source: Front Psychiatry. 2024 Oct 7;15:1459633. doi: 10.3389/fpsyt.2024.1459633 (PMC11491562; doi:10.3389/fpsyt.2024.1459633)
Supplement: Supplementary file 1 [file DataSheet1.docx]

Supplementary Material

# Supplementary Figures and Tables

## Supplementary Table 1. Covariates, ranked by expert medical opinion, used for PS IPW and logistic regression model adjustment comparison

| Rank | Covariate | **Categorisation** | **Notes** |
| --- | --- | --- | --- |
| 1 | Total number of failures in current MDE | 2/3/≥4 | Based on MGH-ATRQ |
| 2 | Age, years | <30/30–44/45–64/≥65 | Data cut-off for ‘elderly patients’ at ≥65 years, other cut-offs determined by exploratory analysis |
| 3 | MADRS score, baseline | <31/31–34/>34 | Data cut-off >34 to identify patients with severe TRD,^1^ cut-off of <31 determined by exploratory analysis |
| 4 | SDS score, baseline | 1–19/20–26/27–30 | Functional impairment is based on baseline SDS total score: not impaired (0–3), mild  (4–11), moderate (12–19), marked (20–26) or extreme (27–30). In this analysis, the categories of not impaired, mild and moderate were pooled together, while marked and extreme were considered individual categories. |
| 5 | Total number of MDE | 1/2–5/6–10/>10 | Based on medical history |
| 6 | Duration of current MDE, weeks | ≤32/33–51/52–103/≥104 | Based on medical history; data cut-offs of 33 and 52 weeks correspond to quartiles of distribution, cut-off of 104 weeks based on SUSTAIN-2 study protocol, which stated ‘if single episode MDD, the duration must be ≥2 years’ |
| 7 | Gender | Male/Female |  |
| 8 | Prior failure on augmentation | No/Yes | Based on MGH-ATRQ |
| 9 | History of suicidality | No event/Suicidal ideation/Suicidal behaviour/Missing | Based on C-SSRS (lifetime) |
| 10 | Time since first diagnosis of MDD, years | <5/5–19/≥20 | Based on medical history; data cut-offs determined by exploratory analysis |
| 11 | Age at diagnosis of MDD, years | <35/35–54/≥55 | Based on medical history; data cut-off of 55 years corresponds to previously conducted post-hoc analysis of ‘late onset’ patients from TRANSFORM-3, cut-off of 35 years determined by exploratory analysis |
| 12 | Prior failure on SSRI | No/Yes | Based on MGH-ATRQ |
| 13 | Prior failure on SNRI |  |  |
| 14 | Prior failure on TCA |  |  |
| 15 | Prior failure on other AD^a^ |  |  |
| 16 | Average duration of each treatment line during current MDE, weeks | <12/12–23/24–51/≥52 | Every patient received multiple treatment lines during their current MDE, these data represent average duration of each individual treatment line and not overall duration of current MDE, data were calculated as duration of current MDE (based on medical history) divided by total number of failures in current MDE (based on MGH-ATRQ); data cut-offs correspond to quartiles of distribution |
| 17 | CGI-S score, baseline | 1–4/5/6–7/Missing |  |
| 18 | EQ-VAS score, baseline | <30/≥30/Missing |  |

All scored covariates measured at baseline. ^a^Prior failure on other AD included trazodone, nefazodone, vilazodone, bupropion, mirtazapine, mianserin, opipramol, agomelatine, tianeptine, reboxetine and vortioxetine. AD: antidepressant; CGI-S: Clinical Global Impression-Severity; C-SSRS: Columbia-Suicide Severity Rating Scale; EQ‑VAS:‍ EuroQoL‑visual analogue scale; IPW: inverse probability weighting; MADRS: Montgomery-Åsberg Depression Rating Scale; MDD: major depressive disorder; MDE:‍ major depressive episode;
MGH-ATRQ: Massachusetts General Hospital Antidepressant Treatment Response Questionnaire; PS: propensity score; SDS: Sheehan Disability Score; SNRI: serotonin-norepinephrine reuptake inhibitor; SSRI: selective serotonin reuptake inhibitor; TCA: tricyclic antidepressant; TRD: treatment resistant depression.

## Supplementary Figure 1. Study flow diagrams for patients included in ICEBERG

## SUSTAIN-2 (esketamine NS)
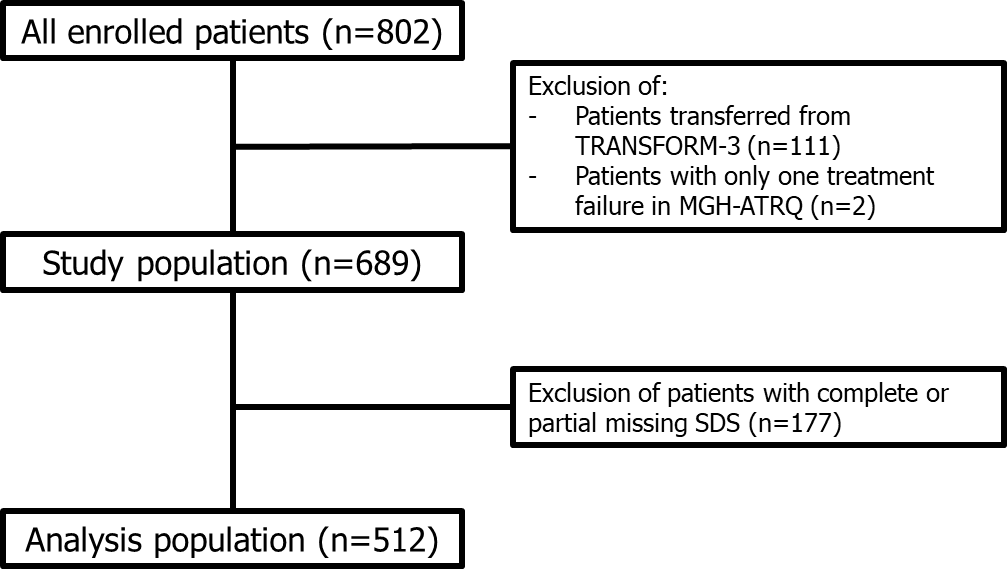


## EOTC (RWT)
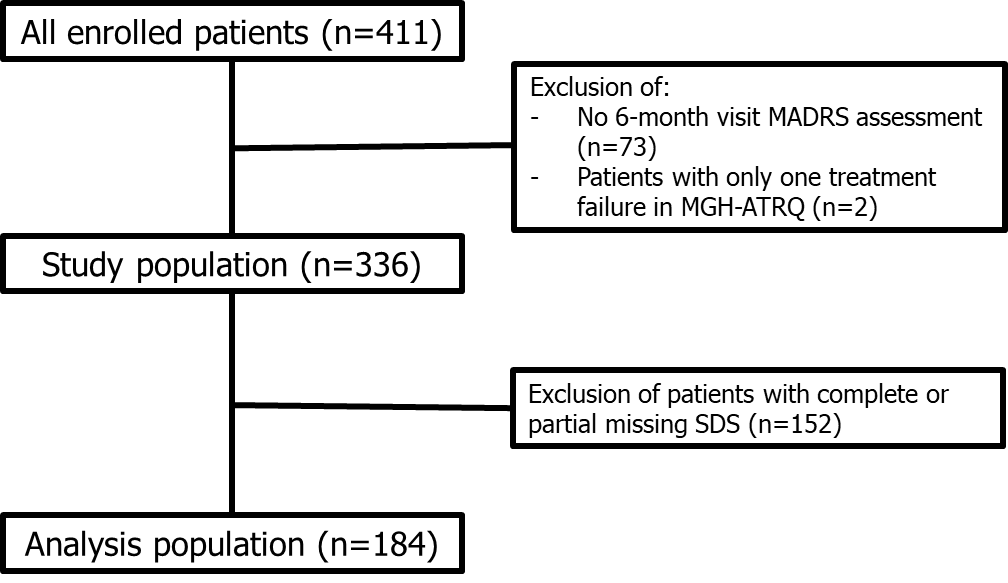


Patient disposition in SUSTAIN-2 (**A**) and EOTC (**B**). Study population refers to the population analysed in the primary and polypharmacy ICEBERG analyses;^2, 3^ Analysis population refers to the population for whom functional remission could be evaluated at 6 months. EOTC: European Observational TRD Cohort; MADRS: Montgomery‑Åsberg Depression Rating Scale; MGH-‍ATRQ:‍ Massachusetts General Hospital Antidepressant Treatment Response Questionnaire; NS: nasal spray; SDS: Sheehan Disability Scale; TRD:‍ treatment resistant depression.

## Supplementary Table 2. SMDs before and after reweighting

| Rank | Covariate | **SMDs before reweighting** | **SMDs after reweighting** |
| --- | --- | --- | --- |
| 1 | Total number of failures in current MDE | 0.147 | 0.228 |
| 2 | Age, years | 0.390 | 0.131 |
| 3 | MADRS score, baseline | 0.224 | 0.156 |
| 4 | SDS score, baseline | 0.162 | 0.063 |
| 5 | Total number of MDE | 0.292 | 0.176 |
| 6 | Duration of current MDE, weeks | 0.274 | 0.170 |
| 7 | Gender | 0.089 | 0.113 |
| 8 | Prior failure on augmentation | –0.022 | –0.038 |
| 9 | History of suicidality | 0.698 | 0.193 |
| 10 | Time since first diagnosis of MDD, years | 0.214 | 0.312 |
| 11 | Age at diagnosis of MDD, years | 0.262 | 0.143 |
| 12 | Prior failure on SSRI | –0.140 | –0.006 |
| 13 | Prior failure on SNRI | –0.112 | 0.172 |
| 14 | Prior failure on TCA | –0.211 | –0.010 |
| 15 | Prior failure on other AD^a^ | 0.028 | –0.009 |
| 16 | Average duration of each treatment line during current MDE, weeks | 0.302 | 0.208 |
| 17 | CGI-S score, baseline | 0.109 | 0.231 |
| 18 | EQ-VAS score, baseline | 0.161 | 0.048 |

^a^Prior failure on ‘other’ included trazodone, nefazodone, vilazodone, bupropion, mirtazapine, mianserin, opipramol, agomelatine, tianeptine, reboxetine and vortioxetine. AD: antidepressant; CGI‑S: Clinical Global Impressions‑Severity; EuroQoL: European Quality of Life; MADRS: Montgomery‑Åsberg Depression Rating Scale; MDE: major depressive episode; NS: nasal spray; RWT: real‑world treatment; SD:‍ standard deviation; SDS: Sheehan Disability Scale; SMD: standardised mean difference; SNRI: ‍serotonin‑norepinephrine reuptake inhibitor; SSRI: selective serotonin reuptake inhibitor; VAS: visual analogue scale.

## Supplementary Figure 2. Distribution of propensity scores by treatment

## Before reweighting
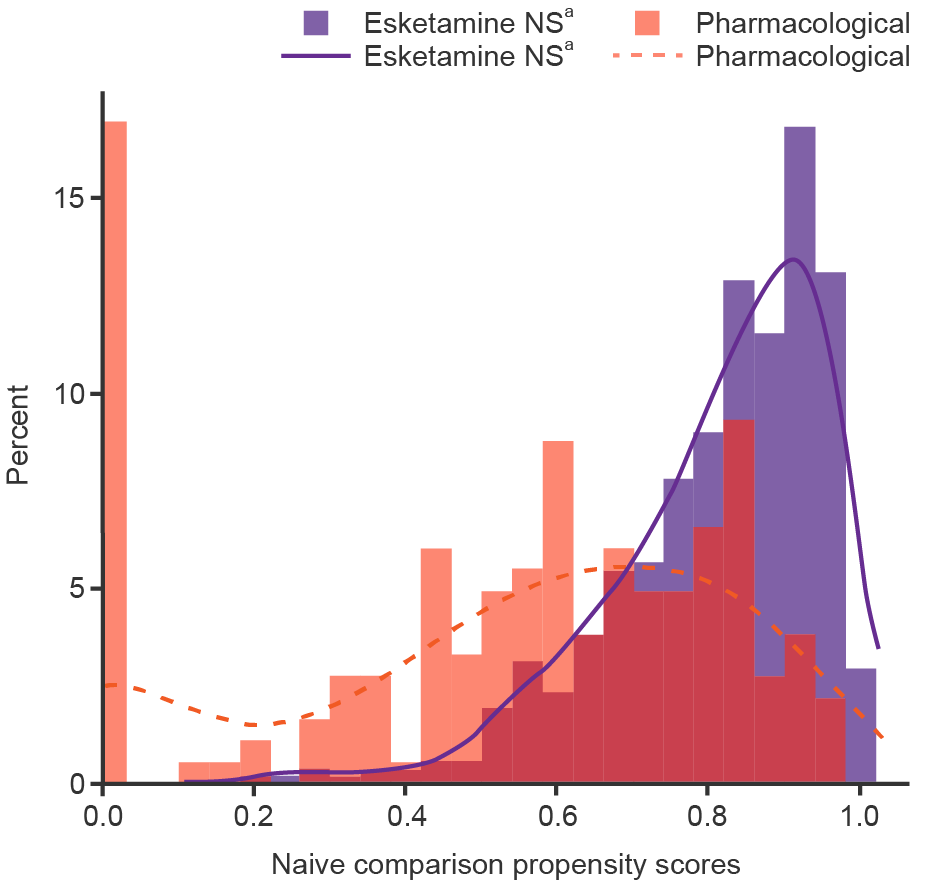


## After reweighting
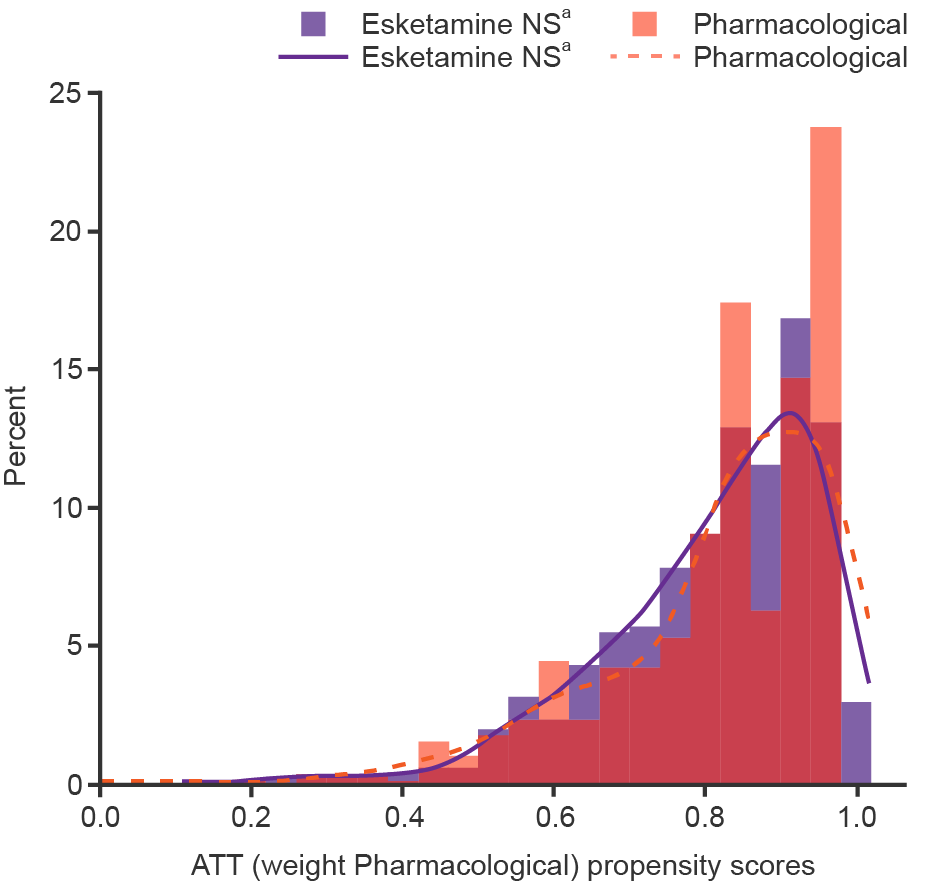


Naïve (A) and reweighted (B) distributions of propensity scores. ^a^Esketamine NS in addition to an SSRI/SNRI. ATT: rescaled average treatment effect among treated; NS: nasal spray; RWT: real‑world treatment; SNRI: serotonin-norepinephrine reuptake inhibitor; SSRI: selective serotonin reuptake inhibitor.

# References

1. Heerlein K, Perugi G, Otte, C, Frodl, T, Degraeve, G, Hagedoorn, W, Oliveira-Maia, AJ, Perez Sola, V, Rathod, S, Rosso, G, Sierra, P, Malynn, S, Morrens, J, Verrijcken, C, Gonzalez, B, Young, AH. Real world evidence from a European cohort study of patients with treatment resistant depression: Treatment patterns and clinical outcomes. Journal of Affective Disorders 2021;290:334–344.

2. Oliveira-Maia AJ, Morrens J, Rive B, et al. ICEBERG study: an indirect adjusted comparison estimating the long-term benefit of esketamine nasal spray when compared with routine treatment of treatment resistant depression in general psychiatry. Frontiers in Psychiatry 2023;14.

3. Oliveira-Maia AJ, Rive B, Morrens J, et al. Indirect adjusted comparison of 6-month clinical outcomes between esketamine nasal spray and other real-world polypharmacy treatment strategies for treatment resistant depression: results from the ICEBERG study. Frontiers in Psychiatry 2023;14.
